# Supplementary material for: Modeling Bi-modality Improves Characterization of Cell Cycle on Gene Expression in Single Cells
Source: PLoS Comput Biol. 2014 Jul 17;10(7):e1003696. doi: 10.1371/journal.pcbi.1003696 (PMC4102402; doi:10.1371/journal.pcbi.1003696)
Supplement: Figure S1 — Separation of asynchronously cycling cells into three cell cycle phase populations (G0/G1, S and G2/M) via fluorescence activated cell sorting (FACS). H9 (A), MB-231 (D) and PC3 (G) cells were sorted based on DNA content as determined via retention of Hoechst 33342 dye. Individual cells were gated on based on forward scatter versus side scatter for H9 (B), MB-231 (E) and PC3 (H) populations. The number and percentage of H9 (C), MB-231 (F) and PC3 (I) cells in a given phase within the asynchronous population as determined by FACS analysis. (PDF) [file pcbi.1003696.s001.pdf]

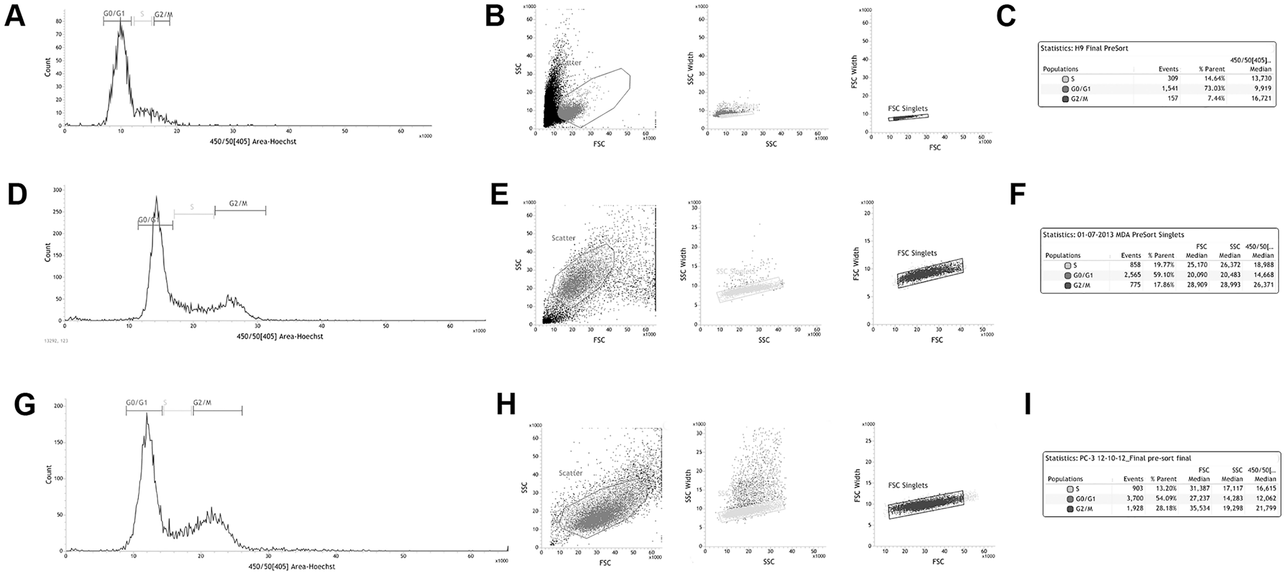
A
G0/G1
S
G2/M
Count
450/50[405] Area-Hoechst
B
SSC
FSC
Scatter
SSC Width
SSC
FSC Width
FSC
FSC Singlets
C
Statistics: H9 Final PreSort
Populations | Events | % Parent | 450/50[405]... Median
S | 309 | 14.64% | 13,730
G0/G1 | 1,541 | 73.03% | 9,919
G2/M | 157 | 7.44% | 16,721
D
G0/G1
S
G2/M
Count
450/50[405] Area-Hoechst
13292, 123
E
Scatter
SSC
FSC
SSC Width
SSC Singlets
SSC
FSC Width
FSC Singlets
FSC
F
Statistics: 01-07-2013 MDA PreSort Singlets
Populations | Events | % Parent | FSC Median | SSC Median | 450/50[... Median
S | 858 | 19.77% | 25,170 | 26,372 | 18,988
G0/G1 | 2,565 | 59.10% | 20,090 | 20,483 | 14,668
G2/M | 775 | 17.86% | 28,909 | 28,993 | 26,371
G
G0/G1
S
G2/M
Count
450/50[405] Area-Hoechst
H
Scatter
SSC
FSC
SSC Width
SSC
FSC Width
FSC Singlets
FSC
I
Statistics: PC-3 12-10-12_Final pre-sort final
Populations | Events | % Parent | FSC Median | SSC Median | 450/50[... Median
S | 903 | 13.20% | 31,387 | 17,117 | 16,615
G0/G1 | 3,700 | 54.09% | 27,237 | 14,283 | 12,062
G2/M | 1,928 | 28.18% | 35,534 | 19,298 | 21,799
