# Supplementary figures and images for: Modeling Bi-modality Improves Characterization of Cell Cycle on Gene Expression in Single Cells
Source: PLoS Comput Biol. 2014 Jul 17;10(7):e1003696. doi: 10.1371/journal.pcbi.1003696 (PMC4102402; doi:10.1371/journal.pcbi.1003696)

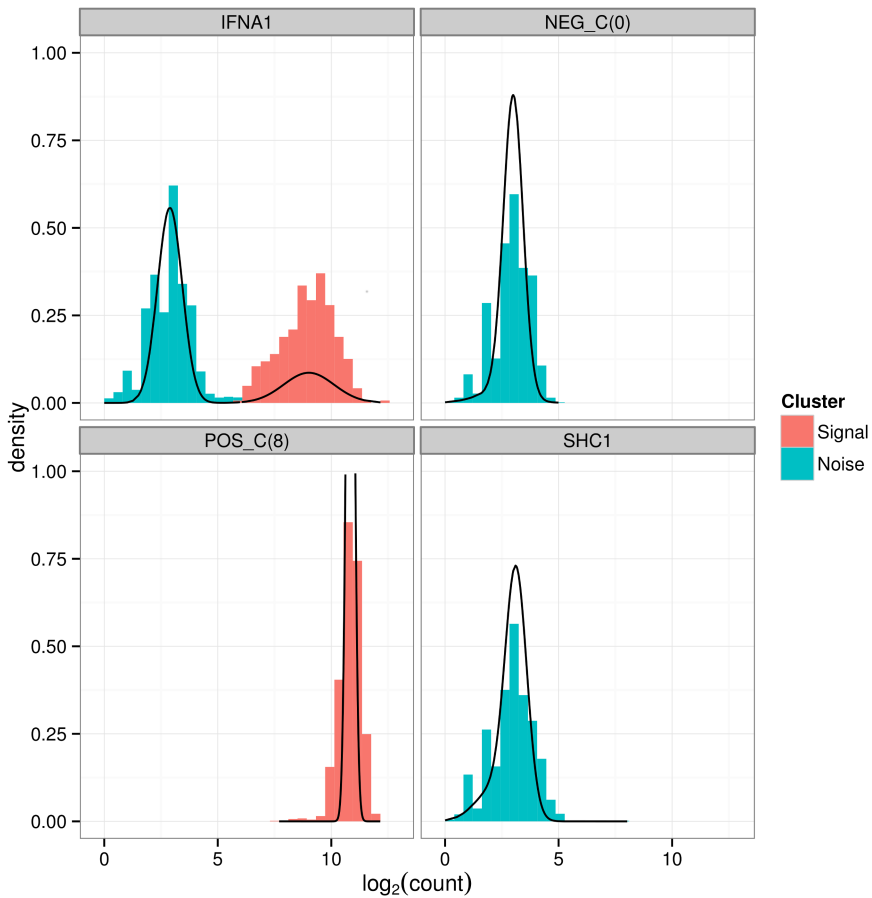

Supplement: Figure S2 — Histogram of log Counts of mRNA for various controls and genes. Positive control primers (for which 100% expression is expected), negative control primers (for which no expression is expected) and two genes with different expression frequencies are shown. The estimated Gaussian mixture densities from the Empirical Bayes model are superimposed. (PDF) [file pcbi.1003696.s002.pdf]

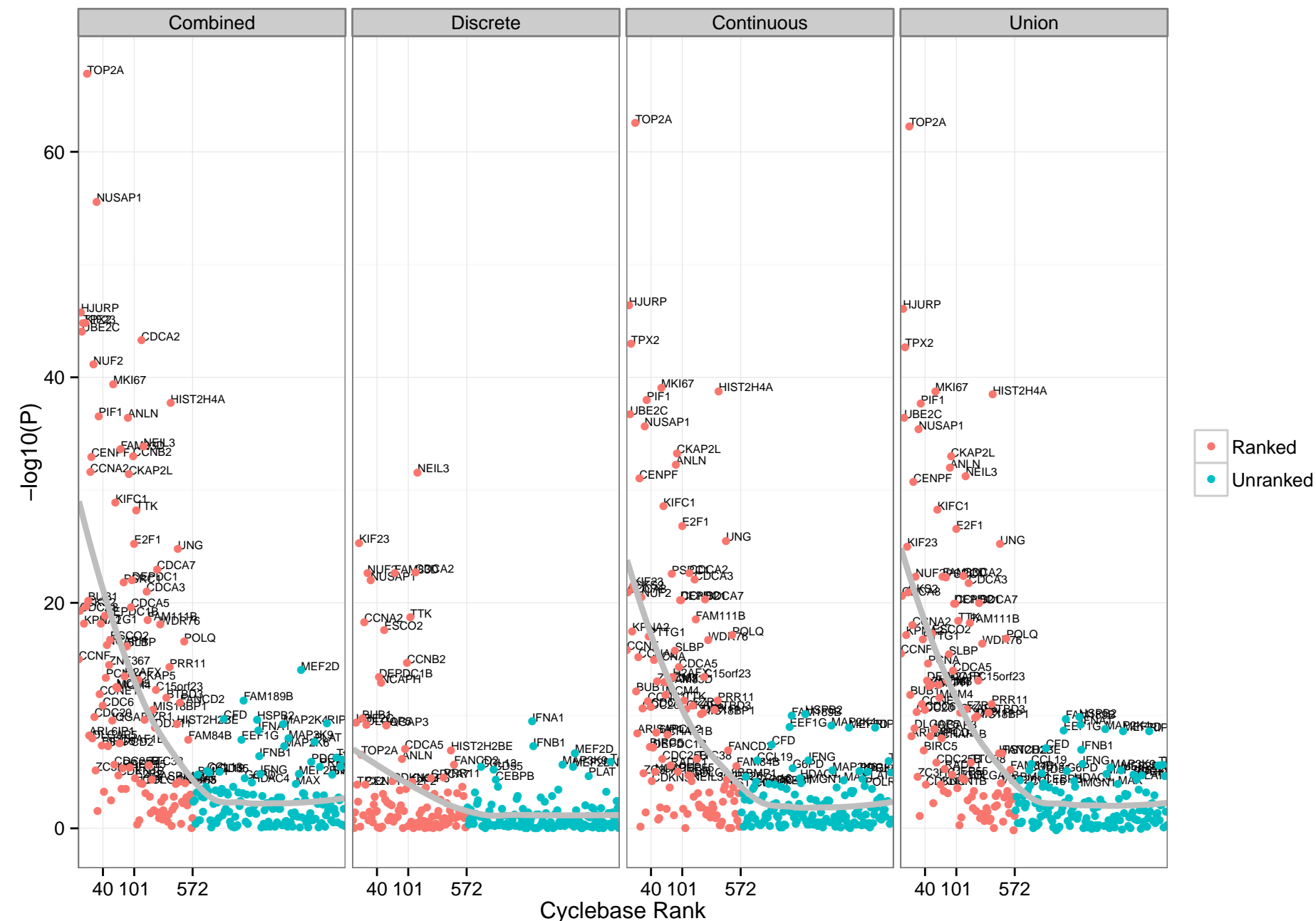

Supplement: Figure S3 — P values testing for differential expression in the Hurdle model decompose into discrete and continuous portions, and a union-intersection on the parameter set. Grey lines indicate average (loess smoothed) P-value for a given gene rank. Both discrete and continuous components offer information about differential expression, and combining them via the Hurdle model offers more sensitive detection of ranked genes compared to a union-intersection test. (PDF) [file pcbi.1003696.s003.pdf]

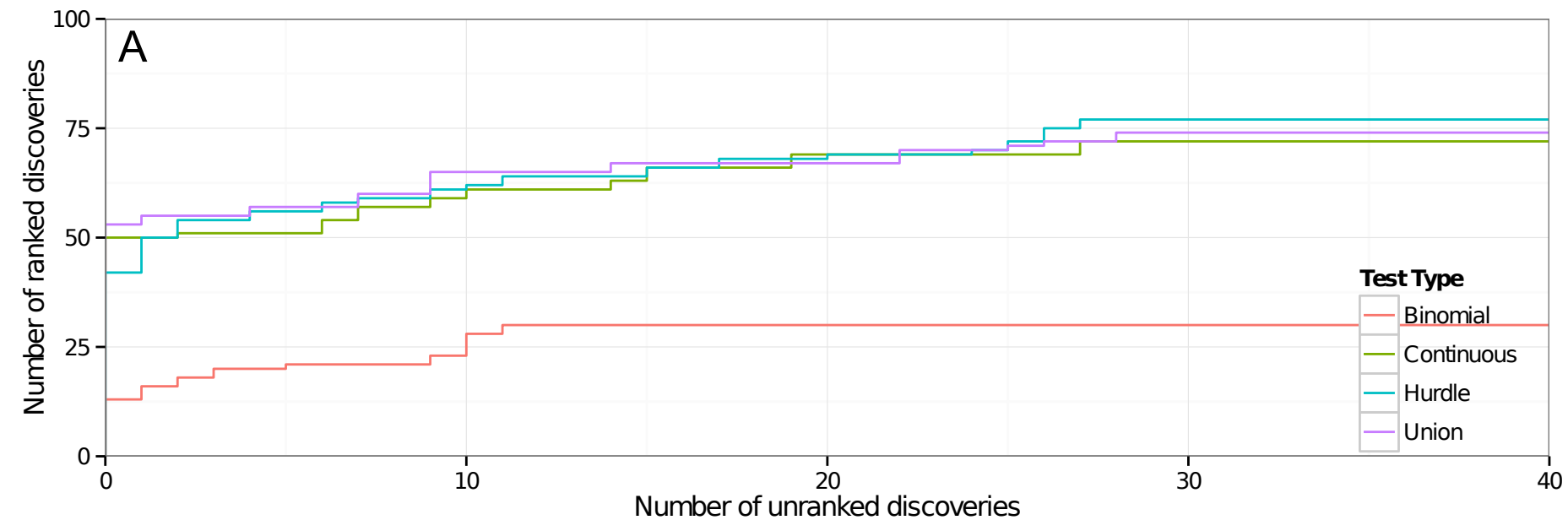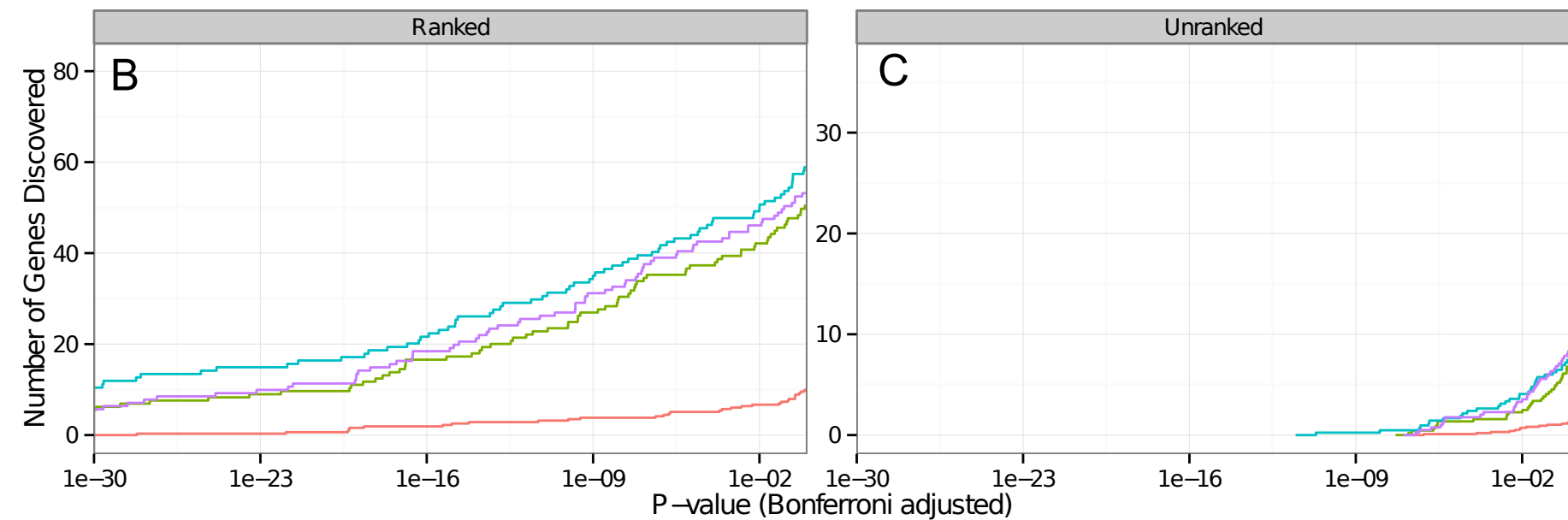

Supplement: Figure S4 — Pseudo ROC plot (A) and number of discoveries versus Bonferroni-adjusted P values for ranked (B) and unranked (C) genes. In panel A the number of discoveries in ranked genes is plotted against the number of discoveries in unranked genes as the level of the test varies. A discovery in a ranked gene, as it has been previously found to be cell-cycle regulated, is more biologically plausible than a discovery in an unranked gene, so the number discovered at a given level is plausibly related to the sensitivity of a test. Likewise, the number of discoveries in unranked genes may be plausibly related to the specificity of the test. In panels B and C the absolute number of discoveries in ranked and unranked gene sets are plotted for various P-values. In both panels, the binomial model uses logistic regression on dichotomized expression values, while the continuous model uses only values with positive expression. All models adjust for cell line and pre-amplification efficiency. The Hurdle, Union and continuous tests are largely equivalent when judged by their area under the curve of the panel A; however the Hurdle is more sensitive than the continuous or union when judged by absolute number of discoveries in panel B. (PDF) [file pcbi.1003696.s004.pdf]

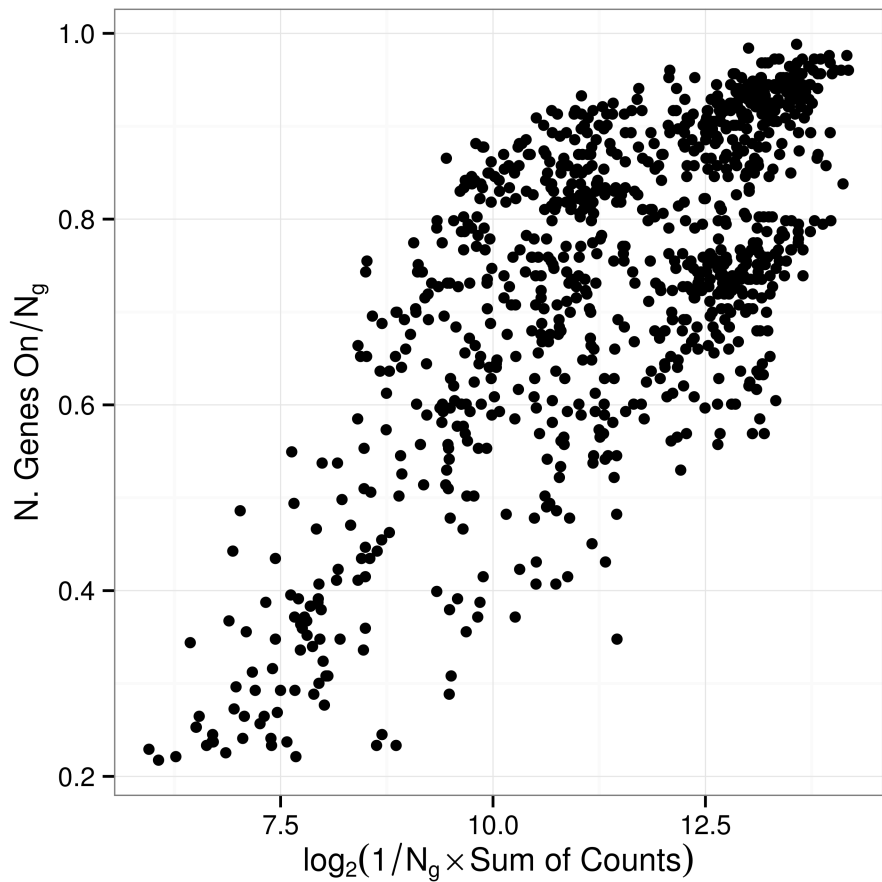

Supplement: Figure S5 — The proportion of expressed genes is related to the log-sum of expression in each cell in our panel of Ng = 253 genes. (PDF) [file pcbi.1003696.s005.pdf]
